# Protozoacidal Trojan-Horse: Use of a Ligand-Lytic Peptide for Selective Destruction of Symbiotic Protozoa within Termite Guts

**DOI:** 10.1371/journal.pone.0106199

**Published:** 2014-09-08

**Authors:** Amit Sethi, Jennifer Delatte, Lane Foil, Claudia Husseneder

**Affiliations:** Department of Entomology, Louisiana State University Agricultural Center, Baton Rouge, Louisiana, United States of America; International Atomic Energy Agency, Austria

## Abstract

For novel biotechnology-based termite control, we developed a cellulose bait containing freeze-dried genetically engineered yeast which expresses a protozoacidal lytic peptide attached to a protozoa-recognizing ligand. The yeast acts as a ‘Trojan-Horse’ that kills the cellulose-digesting protozoa in the termite gut, which leads to the death of termites, presumably due to inefficient cellulose digestion. The ligand targets the lytic peptide specifically to protozoa, thereby increasing its protozoacidal efficiency while protecting non-target organisms. After ingestion of the bait, the yeast propagates in the termite's gut and is spread throughout the termite colony via social interactions. This novel paratransgenesis-based strategy could be a good supplement for current termite control using fortified biological control agents in addition to chemical insecticides. Moreover, this ligand-lytic peptide system could be used for drug development to selectively target disease-causing protozoa in humans or other vertebrates.

## Introduction

One of the most important scientific achievements of the twentieth century has been the development of rapid and effective methods to control insect pests, principally through the use of chemical insecticides. However, the demand for new strategies has been growing due to an increasing recognition of the limitations associated with the use of chemical insecticides, such as insecticide resistance, concerns over environmental and human health impacts, and economic burdens. Therefore, biological control strategies that exploit insect-microbial relationships have been proposed as an alternative to chemical insecticides. The role of microbes in insects as well as the potential use of these microbes and their metabolic capabilities as biological control agents is well documented [1]. However, use of microbes as biological control agents has not been successful for some social insect systems mainly due to the presence of a suite of highly efficient synergistic defense mechanisms against entomopathogens, including behavioral responses (avoidance of pathogen and grooming), antimicrobial compounds, immunity, and competitive endogenous microbial fauna [2]. Thus, precise genetic manipulation to enable microbes that are not recognized as pathogens to interfere with host fitness has been identified as a novel tool to design more efficient biological control agents [2].

The use of genetically altered microorganisms to deliver gene products into a host organism is termed paratransgenesis. Specifically, in insects, genetically engineered microbes capable of colonizing the insect gut could be utilized as “Trojan-Horses” to produce effector molecules that kill the insect pest or eliminate the capacity of insects to act as vectors to transmit pathogenic agents [3], [4]. Paratransgenesis (using genetically engineered bacteria, viruses or fungi) has been predominantly applied to prevent insects from transmitting pathogenic diseases [4]–[12]; only a few studies have used this biotechnology to actually kill the host, i.e. for insect control [13], [14].

One of the major challenges in developing an efficient paratransgenesis system for insect control is the identification of mechanisms that allow microbes to spread efficiently among individuals. This challenge is easily overcome in social insects, such as termites, because they naturally exchange microbes among colony mates via social interactions, including trophallaxis (food exchange), coprophagy, and grooming [14], [15]. Therefore, termites are ideal candidates for the development and application of a paratransgenesis model system for insect pest control [14]–[16].

Design of a control strategy using paratransgenesis requires identification of specific targets, and peptides with toxic effects against the identified target [17], [18]. Subterranean termites are one of the most destructive urban and agricultural pests worldwide. The worker termites, which are responsible for foraging and feeding the colony, harbor cellulose-digesting protozoan symbionts in their hindguts [19]. Disruption of this obligate relationship has dramatic effects on the lifespan of individual termites and the entire colony, as termites deprived of their protozoa die presumably due to inefficient lignocellulose digestion. Thus, the protozoa are suitable targets for designing a paratransgenic system for termite control. Lytic peptides are a ubiquitous part of the non-specific eukaryotic immune system that destroys the integrity of protozoa membranes by disruption or pore formation by wedge-shaped insertion of monomers of the lytic peptide [20]–[22]. Lytic peptides have been shown to kill protozoan parasites in vertebrates [22], [23] but have not been reported to harm the cell membranes of higher eukaryotes [22]–[24].

Recently, Husseneder and Collier [14] used lytic peptides to design a prototype of paratransgenesis for termite control using the Formosan subterranean termite (*Coptotermes formosanus*) as a model. First, they showed that lytic peptides (*Hecate*, *Cecropin*, and *Mellitin*) efficiently killed the three species of protozoa, *Pseudotrichonympha grassii*, *Holomastigotoides hartmanni*, and *Spirotrichonympha leidyi*, associated with the hindgut of *C*. *formosanus* workers. Furthermore, Husseneder and Collier [14] genetically engineered yeast (*Kluyveromyces lactis*) to express *Hecate*. After the yeast was ingested by termite workers, the lytic peptides expressed by the yeast killed the gut protozoa within 4 weeks, followed by the death of the termites within 6 weeks.

The top challenge in developing a paratransgenesis system is to enhance the efficiency of the technology while at the same time preventing from non-target effects of treatments. Lytic peptides have previously been shown to destroy specific cells (e.g., breast, testicular and prostate cancer cells) when they are conjugated with membrane receptor-recognizing molecules [20], [25], [26]. We followed the same concept and identified protozoa–recognition peptides to construct ligands that bind not only to symbiotic protozoa of *C*. *formosanus* but also symbiotic protozoa of another termite species, *Reticulitermes flavipes*, and free-living protozoa. Next, we genetically engineered the yeast *K. lactis* to express a fusion peptide (*Ligand-Hecate*) that specifically killed protozoa. Finally, we developed a target-specific bait containing genetically engineered yeast to kill termites.

## Results

### Identification of ligands that attach to protozoa

**Figure 1:**
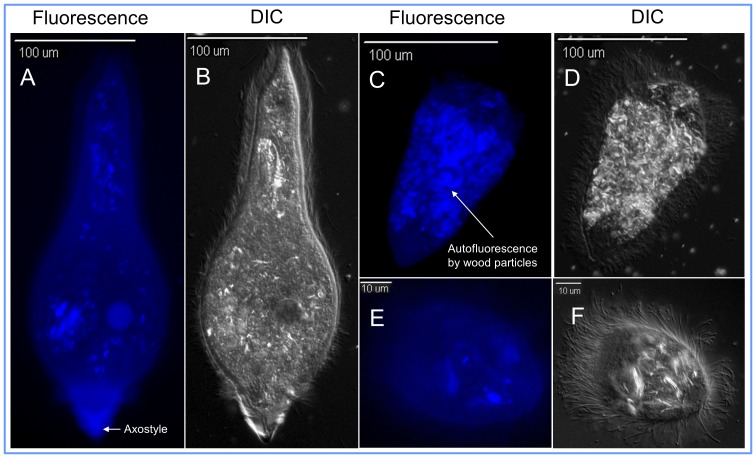
Visualization of binding of fluorescent *Ligand-1* to gut protozoa of the Formosan subterranean termite, *Coptotermes formosanus*. Blue fluorescence (excitation  =  341 nm, emission  =  471 nm) confirms that *Ligand-1* binds to all the three species of the termite protozoa. Phagocytosed wood particles within the protozoa cytoplasm show some patchy autofluorescence. (*A, B*) Fluorescent and differential interference contrast (DIC) exposures of *Pseudotrichonympha grassii*, respectively. Binding of ligands was concentrated in the anterior region of *P. grassii* clearly showing the axostyle (a sheet of microtubules). (*C, D*) Fluorescent and DIC exposures of *Holomastigotoides hartmanni*, respectively. (*E, F*) Fluorescent and DIC exposures of *Spirotrichonympha leidyi*, respectively. Binding of *Ligand-2* also showed a similar fluorescence pattern.

**Figure 2:**
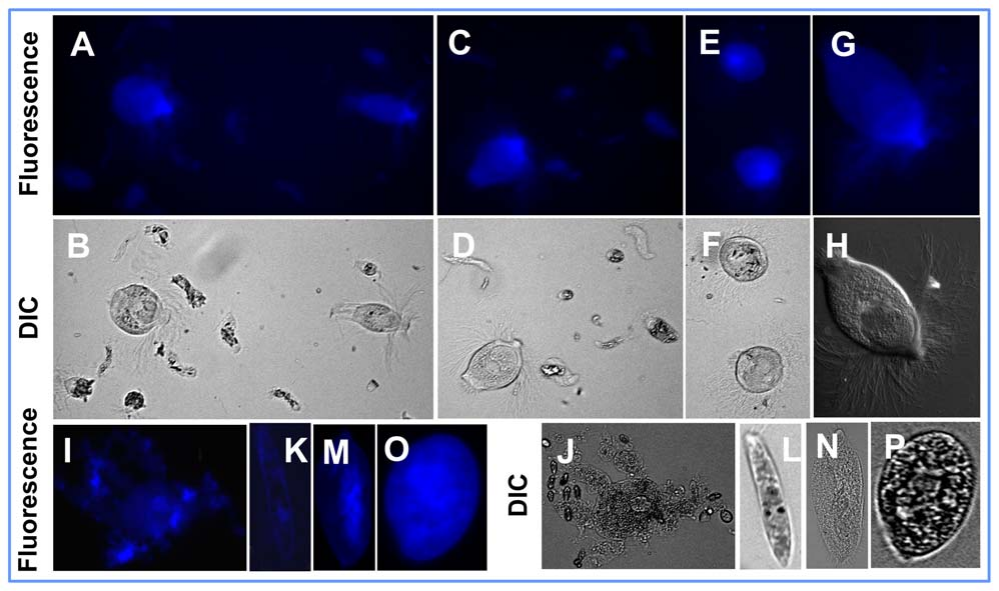
Visualization of binding of fluorescent *Ligand-1* to other groups of protozoa: (*A*–*H*) twelve species of gut protozoa from the eastern subterranean termite, *Reticulitermes flavipes*, and (*I*–*P*) four species of free-living aerobic protozoa. (*I, J*) Fluorescent and differential interference contrast (DIC) exposures of *Amoeba* sp., respectively. (*K, L*) Fluorescent and DIC exposures of *Euglena* sp., respectively. (*M,N*) Fluorescent and DIC exposures of *Paramecium* sp., respectively. (*O, P*) Fluorescent and DIC exposures of *Tetrahymena pyriformis*, respectively. Blue fluorescence confirms that *Ligand-1* binds to all the protozoa tested. Binding of *Ligand-2* also showed a similar fluorescence pattern.

We used a phage library expressing variants of linear random heptapeptides to identify termite protozoa-recognizing peptides (Fig. S1A–G). Nineteen unique heptapeptide sequences that bound to protozoa were identified (Table S1). Two ligands, ALNLTLH (*Ligand-1*) and LPSLPAN (*Ligand-2*) showed homology to epitopes present on the variant surface glycoprotein (VSG) of *Trypanosoma brucei* and a single-pass type II membrane protein of *Thermosynechococcus elongatus*, respectively, when searched in the Database of Interacting Proteins (DIP, http://dip.doe-mbi.ucla.edu/). As it was not feasible to test all 19 selected candidate ligand peptide in our study, we selected *Ligand-1* and *Ligand-2* for synthesis based upon their predicted interactions described above. Next, the ligands were attached to the fluorophore *EDANS* (5-((2-Aminoethyl) amino) naphthalene-1-sulfonic acid) (Fig. S1H,I) to confirm their specific binding to termite protozoa under *in vitro* (protozoa culture) and *in vivo* (termite enema) conditions. Both of the ligands bound to all three species of protozoa of *C*. *formosanus* and not to the termite hindgut wall (Fig. 1). The ligands bound to the entire surface of protozoa, but were mostly concentrated in the anterior region of *P. grassii* clearly showing the axostyle (a sheet of microtubules) (Fig. 1A). For untreated protozoa, we only observed some patchy autofluorescence of wood particles ingested by the protozoa. However, the autofluorescence was easy to distinguish from specific binding of the ligands, since there is no autofluorescence of the surface and the axostyle region (Fig. S2A–C) These binding sites are likely to be present in all species of protozoa, as both the ligands also bound to all eleven species of protozoa [27] found in the hindgut of another termite species *Reticulitermes flavipes* (Fig. 2A–H) and the four free-living aerobic protozoa species tested (*Tetrahymena pyriformis*, *Amoeba* sp., *Euglena* sp., and *Paramecium* sp.) (Fig. 2I–P). The ligands are most likely protozoa-specific as they did not bind to non-target microorganisms, such as gram negative *Escherichia coli*, gram positive *Pilibacter termitis* (a lactic acid bacterium exclusively found in the gut of *C*. *formosanus*) [28] and the yeast *K*. *lactis*.

### Addition of ligand increases target specificity and efficiency of lytic peptides

**Figure 3:**
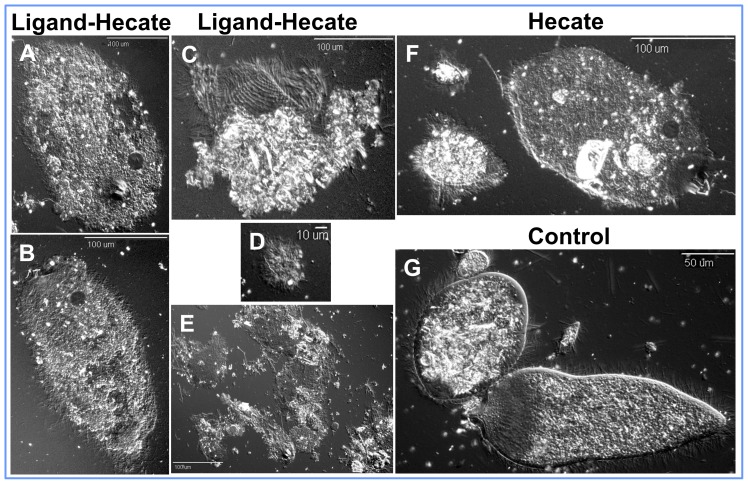
Enhanced toxicity of *Ligand-Hecate* fusion peptide compared to *Hecate* alone. Membranes of the termite protozoa lose their integrity five-fold faster when incubated with *Ligand-Hecate* fusion peptide as compared to incubation with *Hecate* alone at 1 µM concentration. (*A, B*) Differential interference contrast (DIC) images of *P. grassii* after 5 min of incubation with *Ligand-Hecate*. (*C*) DIC image of *H*. *hartmanni* after 5 min of incubation with *Ligand-Hecate*. (*D*) DIC image of *S. leidyi* after 5 min of incubation with *Ligand-Hecate*. (*E*) DIC images of all the three species of termite protozoa after 10 min of incubation with *Ligand-Hecate*. (*F*) DIC image of all the three species of termite protozoa after 10 min of incubation with *Hecate* alone. (*G*) DIC image of all the three species of termite protozoa after 10 min of incubation with the buffer without any peptide (control).

**Figure 4:**
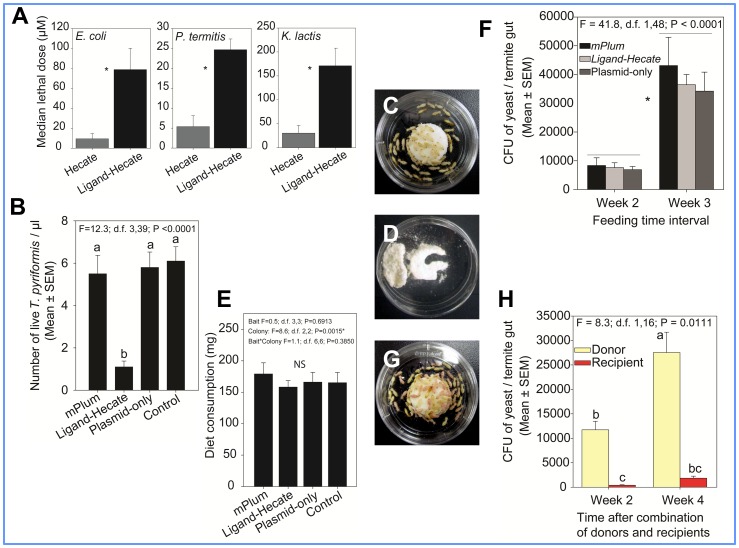
Assays using *Ligand*-*Hecate* and *Hecate* peptides, and genetically engineered yeast strains. (*A*) Mean lethal doses (LD_50_) of *Ligand*-*Hecate* and *Hecate* peptides against non-target microorganisms *Escherichia coli*, *Pilibacter termitis*, and *Kluyveromyces lactis*. The linking of *Ligand-1* with *Hecate* significantly enhanced the mean lethal dose for each non-target microorganism. * indicates significant difference between treatments. (*B*) Toxicity of culture supernatants of different yeast strains against aerobic protozoa *T*. *pyriformis*. (*C*) Termite workers feeding on α-cellulose bait disk containing genetically engineered yeast cells in a bioassay setup. (*D, E*) Bait consumed by termite workers after five weeks. Addition of yeast into α-cellulose matrix did not deter termites from feeding and no significant difference was found in the diet consumption among different treatments. (*F*) Increasing number of yeast cells in the termite gut at two and three weeks of ingesting α-cellulose bait containing genetically engineered yeast strains. Control bait containing only α-cellulose did not show any CFU of *K*. *lactis*. (*G*) Bioassay setup to test transfer of the genetically engineered yeast cells to other nestmates. Termites fed on α-cellulose bait containing *mPlum* expressing yeast strain for two weeks (donors) were mixed with an equal number of workers from the same colony that were not fed on yeast bait (recipients, stained red with 1% Sudan Red 7B) and the mixed termites were fed on plain α-cellulose bait without any yeast in a Petri dish. (*H*) Number of *mPlum* expressing yeast cells (CFU) recovered from the donor and recipient termite guts two and four weeks after combining donors and recipients.

Since both fluorescent ligand complexes showed similar binding characteristics, only *Ligand-1* was conjugated to *Hecate* (*Ligand-Hecate*, named hereafter) (Fig. S1J) to confirm its protozoacidal specificity and efficiency. One micromolar solution of *Ligand-Hecate* fusion peptide killed all three species of protozoa of *C. formosanus in vitro* in less than 10 min (Fig. 3A–E). However, the same concentration of *Hecate* alone (without the ligand) required more than 30 min to kill the protozoa (Fig. 3F). Increased efficiency of *Ligand-Hecate* compared to Hecate alone was also confirmed for the four species of free-living aerobic protozoa. Twenty-four hours after injection of *Ligand-Hecate* into the hindgut of *C*. *formosanus* workers via enemas, all three species of protozoa in the hindguts were dead. Treated termites died within two weeks after the loss of their protozoa. Target specificity was further confirmed by incubating non-targets *E*. *coli, P. termitis*, and *K*. *lactis* with *Ligand-Hecate* fusion peptide and *Hecate* alone. Median lethal dose (LD_50_) of *Ligand-Hecate* was 8.3, 4.6 and 5.6-fold significantly higher than *Hecate* when tested against *E*. *coli*, *P. termitis*, and *K*. *lactis*, respectively (Fig. 4A). Thus, the addition of *Ligand-1* to *Hecate* increases not only the protozoacidal efficiency but also prevents immediate lysis of non-target species.

### Termite bait containing protozoa-killing yeast strain

**Figure 5:**
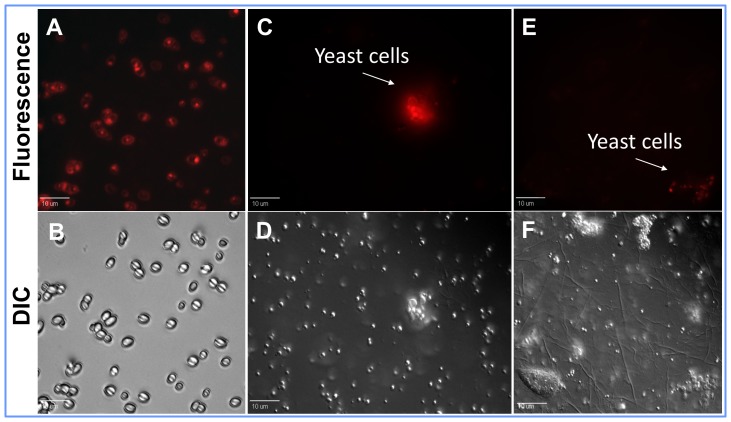
Genetically engineered *Kluyveromyces lactis* yeast expressing the far red fluorescent protein *mPlum* (excitation – 590 nm and emission – 649 nm). (*A*) Fluorescent and (*B*) Differential interference contrast (DIC) images of yeast cells expressing *mPlum* after 48 h of culture, respectively. (*C, E*) Fluorescent and (*D, F*) DIC images of yeast cells expressing *mPlum* inside the termite gut after two weeks of their continuous ingestion, respectively.

After confirming the target specific toxicity of the *Ligand-Hecate* fusion peptide, the commercially available *K. lactis* yeast was genetically engineered to express *Ligand-Hecate*. Simultaneously, another *K. lactis* strain expressing a red fluorescent protein *mPlum* was prepared to monitor ingestion and survival of yeast in the termite's guts, as well as spread of genetically engineered yeast among colony mates. Forty-eight hours old cultures of both the yeast strains secreted *mPlum* and *Ligand-Hecate*, respectively. Both the culture supernatant and pelleted *mPlum* yeast cells showed red fluorescence (Fig. 5 A,B). The culture supernatant from the *Ligand-Hecate* yeast strain caused 82% mortality compared to control in a free-living aerobic protozoa *T. pyriformis* after 24 h of treatment (Fig. 4B). We did not quantify the expression of *Ligand-Hecate* in the supernatant. Thus, the incubation experiment using culture supernatant was used as indirect evidence to suggest that the observed mortality of *T. pyriformis* could possibly be attributed to the *Ligand-Hecate* produced by the yeast.

The freeze-dried yeast strains (expressing *Ligand-Hecate*, *mPlum*, and a control containing only the vector plasmid with no inserted gene) were individually mixed with α-cellulose bait and control α-cellulose bait matrix without any yeast strain and were fed to termite workers (Fig. 4C,D). Addition of yeast in the bait matrix (α-cellulose) did not deter termite feeding and termites consumed similar amounts of bait among treatments (Fig. 4E).

**Figure 6:**
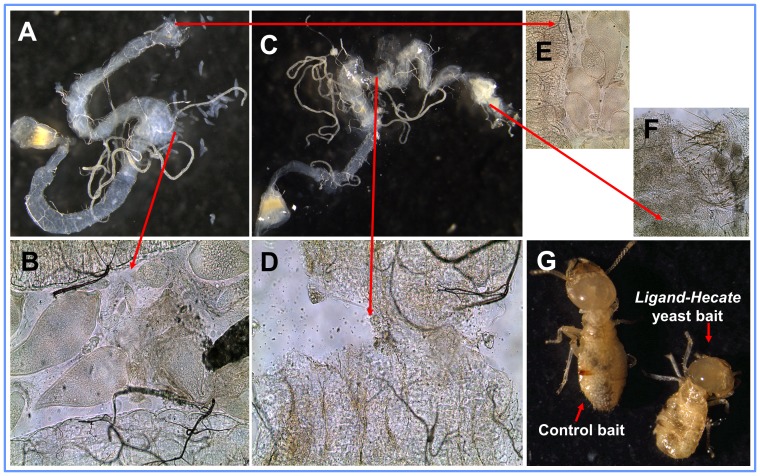
α-cellulose bait containing *Ligand-Hecate* expressing yeast strain kills all the three species of protozoa found in *C*. *formosanus* workers within three weeks and the workers die within five weeks of bait ingestion. (*A*) The gut of a worker with live protozoa at three weeks of ingesting plain α-cellulose bait. (*B*) Healthy protozoa exude out of worker gut when the gut is cut open. (*C, D*) The empty paunch of a worker possessing no protozoa at three weeks of ingesting the bait containing *Ligand-Hecate* yeast. (*E*) Healthy and *(F)* cellular debris of protozoa in the rectum of a worker at three weeks of ingesting the bait containing no yeast and *Ligand-Hecate* yeast, respectively. (*G*) Workers at five weeks of ingesting α-cellulose bait containing no yeast (left) and *Ligand-Hecate* expressing yeast (right). The worker fed on *Ligand-Hecate* bait is dead.

After two weeks of bait consumption(Fig. 4D), we were able to confirm the ingestion of yeast strains by the termites via plating gut contents on *Kluyveromyces* differential medium and observing the growth of yeast colonies with the characteristic blue color. At the same time we also confirmed gene expression of the *mPlum* yeast strain by observing red fluorescence of yeast cells in the termite gut and yeast colonies cultured from gut contents (Fig. 5C–F). The number of yeast cells (counted as colony forming units after culture of gut contents on *Kluyveromyces* differential media) per worker gut significantly increased from the second week to third week after the termites began ingesting the baits containing yeast (Fig. 4F, Table S2). After three weeks of feeding on the bait containing *Ligand-Hecate* expressing yeast, all three species of protozoa were dead and cellular debris of protozoa was found in the rectum of the workers (Fig. 6); all workers died within five weeks of continuous yeast ingestion.

### Termites transfer genetically engineered yeast to nestmates via social interaction

Following visual detection of *mPlum* expressing yeast in termite guts after 2 weeks of ingesting the bait (see above), the remaining workers (donors, i.e. previously fed on mPlum yeast) were combined with an equal number of workers from the same colony that were fed on cellulose without yeast (recipients); the recipients were marked red by fat body stain (Sudan Red 7B) to distinguish them from donors (Fig. 4G). Both the donors and the recipients were fed on plain α-cellulose bait matrix without any yeast strain, and *mPlum* yeast was detected in the recipients at two weeks after both groups were combined. The number of *mPlum* yeast cells (CFU) significantly increased in the donors from the second week to fourth week, even though ingestion of yeast from bait was discontinued when donors and recipients were combined (Fig. 4H, Table S3). The number of yeast cells also increased in the recipients, but the increase was not significant within the measured time span.

## Discussion

Paratransgenesis has been used primarily to control insect vector-borne diseases of humans and agricultural crops, where symbiotic microbes were genetically engineered to deliver molecules that block pathogen transmission [3]–[7], [9], [11], [12], [29]. Here, we provide the first example of a target-specific paratransgenesis system that has the potential to eliminate insect pests. This paratransgenesis system uses a conjugate of recognition and lytic molecules (*Ligand-Hecate*) to kill the cellulose-digesting protozoa in the termite gut. The current findings demonstrate delivery, retention and biological activity of genetically engineered *K*. *lactis* yeast in the gut of Formosan subterranean termites.

The proof of concept of paratransgenesis in termites was first achieved by Husseneder and Grace [15], who genetically engineered *Enterobacter cloacae* isolated from the gut of *C. formosanus* to express ampicillin resistance markers and green fluorescent protein. The engineered bacteria were rapidly ingested by workers, efficiently transferred among nestmates and were detectable in termite guts for up to two months. Subsequently, Zhao et al. [13] genetically engineered *E*. *cloacae* to express insecticidal proteins from the bacterium *Photorhabdus luminescens* to kill termites. However, *E*. *cloacae* is not an ideal organism for paratransgenesis because it is ubiquitous in nature and causes a variety of infections and problems associated with humans. Moreover, insecticidal toxins from *P*. *luminescens* have considerable mammalian toxicity [30], [31]. Thus, we choose *K*. *lactis* yeast and lytic peptides to develop a prototype of paratransgenesis to control termites [14]. The yeast is non-pathogenic for vertebrates and lytic peptides are not known to harm higher eukaryotes [21], [23]. Moreover, our approach is to kill termites indirectly via targeting obligate gut protozoa linked to cellulose digestion and other processes.

To further enhance environmental safety of a termite paratransgenesis system, we designed effector molecules to specifically target the protozoa [3], [4]. We conjugated lytic peptides to protozoa-specific ligands. Based on the database search, the two ligands showed homology to epitopes present on the membrane proteins (peripheral and transmembrane, respectively) that are involved in several trafficking pathways. *Ligand-1* showed homology with VSG of *T*. *brucei*, which is a glycosylphosphatidylinositol-anchored glycoprotein expressed on the external surface of the protozoan at extreme density (∼ five million) [32], [33]. *Ligand-2* showed homology with the biopolymer transport protein (single-pass type II ExbD family), which is involved in the transport of vitamin B_12_, iron siderophores, sucrose, nickel and sulfates [34]–[36]. Since *Ligand-1* showed homology with the epitopes of VSG, it might be possible that *Ligand*-1 binds to a VSG-like protein that is endocytosed in a similar way as described for trypanosomes [37]–[43]. Thus, the binding of *Ligand-Hecate* on a VSG-like protein could have facilitated rapid membrane internalization and could lead to increased protozoacidal activity of the fusion peptide by five-fold over *Hecate* alone. At the same time, the conjugation of *Ligand-1* to *Hecate* increased the LD_50_ of the fusion peptide for non-target microbes by four to eight-fold over *Hecate* alone. Based on the therapeutic research on ligand-lytic peptide conjugates [20], [25], [26], it is possible that fusion of *Ligand*-1 to *Hecate* interferes with *Hecate*'*s* insertion into the cell membrane and thereby decreases its affinity against non-target species. On the other hand in case of termite protozoa, it is possible that fusion of *Ligand*-1 to *Hecate* provides more stability to *Hecate* and exposes *Hecate* molecules in a close proximity of the cell membrane after *Ligand*-1 binds to the membrane receptors. Thus, the conjugation of the lytic peptide to a ligand increases not only the activity but also enhances the selectivity. Similar results have been found in cancer treatment studies using hormone ligand-lytic peptide conjugates [20], [25], [26]. Lytic peptides are required in less than one micromolar range to effectively kill protozoa [44]; the linking of ligand to lytic peptide even further reduces the minimal activity range. Development of resistance to lytic peptides has not yet been observed, possibly due to the pore-forming mode of action and the rapid environmental degradation that reduces selection pressure [22], [45]. Hence, it appears that ligand–lytic peptide combinations are an ideal effector molecule to specifically kill termite protozoa with low risk to non-target organisms.

Another important feature of a successful paratransgenesis system is uncompromised fitness of the Trojan-Horse in the insect gut [3], [4]. The Trojan-Horse should be able to survive and multiply in the insect gut and further propagate in the insect population. In our studies, we found that the genetically engineered yeast was retained in termite guts and multiplied without continuous feeding on the yeast bait. Further, infected termites transferred the yeast to other nestmates via social interaction and it propagated in recipients. Since two weeks was the first observation time in the transfer experiment, transfer of yeast from infected termites to recipients is likely to occur more rapidly, as Husseneder and Grace [15] previously reported that transfer of bacteria from infected to recipients occurs within hours and even ratios as low as 1 infected termite: 25 recipients were sufficient to spread the bacteria throughout laboratory colonies. Thus, our termite paratransgenesis system using the yeast fulfills the requirement of Trojan-Horse colonization.

In summary, we present evidence for a novel, functional, target-specific and potentially environmentally-friendly termite baiting system with a living agent that expresses a continuous source of effector molecules in the termite colony. Such paratransgenesis-based termite control is attractive due to easy mass production of yeast in bioreactors [46] and relatively easy delivery of the Trojan Horse in the form of baits containing a lyophilized delivery system [47]. Amalgamation of paratransgenic yeast into current termite baiting systems or in conjunction with soil treatments would also likely contribute to enhancing the efficacy of chemical insecticides against termites. Uptake and horizontal transfer of the bait containing the yeast can be further enhanced as demonstrated in chemical insecticide baits by adding known feeding stimulants, such as sugars, amino acids and lipids [48]. Similar to Bt transgenic crops, an additional environmentally-friendly feature can be added to the paratransgenesis system by expressing the effector molecule in inactive form (pro-peptide) [49], [50] that requires activation by digestive proteases that are produced by the protozoa and/or the termite hindgut tissue [51].

Besides termite control, this paratransgenesis biotechnology could be modified for use to control other insect pests that are dependent on symbiotic microbes or to eliminate protozoa in insect vectors. Finally, from a wider perspective, the effector molecule (*Ligand*-*Hecate*) efficiently killed all protozoa species tested and thus could also be used to develop drugs against parasitic protozoa (*Leishmania, Trypanosoma, Trichomonas*, and *Plasmodium*) within vertebrates or invertebrate hosts.

## Materials and Methods

### Termite collection and protozoa isolation

Three colonies of *C*. *formosanus* and one colony of *R*. *flavipes* were collected from New Orleans, Louisiana. The termite species collected herein are not endangered or protected. Thus, no specific permissions were required for the collection of termites. Claudia Riegel, Kenneth Brown and Edward Freytag from New Orleans Mosquito, Termite & Rodent Control Board helped in collecting the termites. After collection, the termites were maintained on damp cardboard in plastic buckets at 26±2°C and 85% R.H. Three groups of 50 worker guts were extirpated from each colony and placed in 100 µl Trager U media (pH 7.0) sparged with gas mixture of nitrogen (92.5%), carbon dioxide (5%) and hydrogen (2.5%) on a glass slide under anaerobic conditions in a glove box (Coy Laboratories Inc., MI, USA) [52]. The hindguts were pierced with a pair of sterile fine dissecting probes to release the protozoa. The gut contents were transferred into a 1 ml microcentrifuge tube containing 900 µl Trager U media. After allowing for sedimentation of gut wall fragments (∼5 sec), the supernatant (900 µl) was transferred into a fresh tube. Then, the protozoa (Fig. S1A–C) were centrifuged at 30×g for 10 min at 4°C. The pellet was collected after rinsing it twice with Trager U.

### Identification of termite protozoa recognition peptides using phage display

We used phage display libraries (Ph.D. 7 Phage Display Peptide Library Kit, New England Biolabs Inc., MA, USA) to identify protozoa recognition peptides by an *in vitro* selection process called panning (Fig. S1G). The pellet (protozoa) was suspended in sparged ice-cold 10 mM Tris-HCl buffer at pH 7.0 that contained 2 mM phenylmethyl sulphonyl fluoride and 2 mM MgCl_2_ [53]. The cells were allowed to swell in the hypotonic buffer for 1 h in an anaerobic chamber. The cells were homogenized and cell breakage was monitored by phase contrast microscopy. The homogenate was layered over a two step gradient consisting of 8 ml of 0.5 M mannitol over 4 ml of 0.58 M sucrose, both in Tris buffer and was centrifuged at 250×g for 30 min. The pellet was resuspended in 3 ml Tris buffer and homogenized again. The second homogenate was layered on a single step gradient that consisted of 20% sucrose in Tris buffer and centrifuged at 250×g for 30 min. The supernatant was collected and centrifuged at 40,000×g for 1 h. The obtained pellet containing plasma membrane was resuspended directly in Tris buffer and stored at −20°C for future use. The purity of the plasma membrane layer was assessed by electron microscopy [54] (Fig. S1D–F).

Isolated plasma membranes were coated on plates and incubated with the phage library as per manufacturer's instruction. After washing of unbound phages, the specifically bound phages were eluted and amplified in *E*. *coli*. Additional 3 rounds of panning were performed to achieve positive selection (Fig. S1G). After positive selection, a pool of ninety phages (10 phages per replication per termite colony) were purified and sequenced to identify the displayed heptapeptide sequences.

### Sequence analysis

The obtained heptapeptide sequences of the phages were compared to those in Genbank using Swissprot: BLAST (http://www.expasy.ch/tools/blast/) for identification of potential protozoa recognition peptides (ligands). A minimum 1000 E-value was used for the search. Next, ligand identity was used in database of interacting proteins (DIP, http://dip.doe-mbi.ucla.edu/) to determine potential binding partners. Ligand sequences were deposited in the NCBI Probe database under Probe Unique Identifiers (PUIDs) 16719496–16719514.

### Conjugation of ligands to fluorophore and lytic peptide

Two heptapeptides were selected out of 19 identified unique sequences to synthesize two ligands *Ligand-1* and *Ligand-2*. Each ligand was coupled to a fluorophore *EDANS* (5-((2-Aminoethyl) amino) naphthalene-1-sulfonic acid) via solid state peptide synthesis using NovaTag resin (EMD Biosciences) at the Louisiana State University peptide facility (Fig. S1H,I) to prepare two Ligand-EDANS complexes (*Ligand-1-EDANS* and *Ligand-2-EDANS*). EDANS can be directly visualized in fluorescence microscopy by the use of an UV light source and a DAPI filter [55]. Since both fluorescent ligand complexes showed similar binding characteristics (see results), only *Ligand-1* out of the two ligands was conjugated to Hecate to prepare ligand-lytic peptide fusion peptide (*Ligand-Hecate*) (Fig. S1J). *Ligand-Hecate* and *Hecate* (without ligand) were synthesized at the Interdisciplinary Center for Biotechnology Research, University of the Florida, USA.

### Testing binding of *Ligand-EDANS* to protozoa, bacteria and yeast

Termite protozoa were isolated as described above and control cultures of the aerobic protozoa *Tetrahymena pyriformis*, *Amoeba* sp., *Euglena* sp., and *Paramecium* sp. (Carolina Biological Supply Company, NC, USA) as well as cultures of *E. coli* and *K*. *lactis* (New England Biolabs Inc., MA, USA) were prepared according to the supplier instructions. Cultures of *Pilibacter termitis* (American Type Culture Collection, KS, USA) were prepared according to the methods given in Higashiguchi et al. [28). All microorganisms were fixed in 10% formaldehyde at 4°C for 12 h [52]. Fixing is necessary to prevent movement of the microorganisms for detailed observation, picture and documentation of fluorescence. In addition, termite protozoa are strictly anaerobic. Without fixing, the fluorescent signal cannot be properly detected as the protozoa cells disrupt due to slight exposure to oxygen during slide preparation for fluorescence microscopy.

For *in vitro* testing, all microorganisms were incubated for 1 h with two *Ligand-EDANS* solutions (*Ligand-1-EDANS* and *Ligand-2-EDANS*), separately at 1 µM final concentration and observed under a fluorescent microscope (excitation  =  341 nm, emission  =  471 nm; Model: DMRxA2, Leica Microsystems Inc.) at 400 × magnification. For *in vivo* testing, each worker was injected into the rectum with 0.3 µl of 1 µM *Ligand-EDANS* solutions using micromanipulators (Leitz micromanipulators, Vermont Optechs Inc., VT, USA) and a pedal-driven high-speed electronic injection system [52]. Control termites were injected with the buffer only. The experiment had three replications with 20 workers in each replication. After injections, the workers were placed into separate Petri dishes with damp filter paper and kept at 26±2°C with 85% R.H. Guts from the injected workers were extirpated after 24 h and the protozoa were collected, fixed and observed.

### Testing toxicity of *Ligand-Hecate* against protozoa, bacteria and yeast

Cultures of all microorganisms were prepared as described above. For *in vitro* testing, termite protozoa were incubated for 1 h with *Ligand-Hecate* solution (end concentration 1 µM). Controls included: (a) protozoa incubated with *Hecate* solution (end concentration 1 µM), and (b) protozoa incubated with the buffer without any peptides. Survival of protozoa was observed after 5, 10, 30 and 60 min of incubation. For *in vivo* testing, each worker was injected into the rectum with 0.3 µl of either (a) 1 µM *Ligand-Hecate* solution, (b) 1 µM *Hecate* solution, or (c) the buffer without any peptide using micromanipulators and a pedal-driven high-speed electronic injection system [52]. The experiment consisted of three replications with 20 workers in each replication. After injections, the workers were placed into separate Petri dishes with damp filter paper and kept at 26±2°C with 85% R.H. Guts from five injected workers were extirpated after 24 h and the protozoa were collected, fixed (as explained above) and observed for mortality. Once the death of protozoa in the termite gut was confirmed, the mortality of the remaining termites was assessed daily.

Cultures of *E. coli, P. termitis* and *K*. *lactis* were incubated for 1 h with six end-concentrations (1, 10, 25, 50, 75 and 100 µM) of *Ligand-Hecate* solution or *Hecate* solution. For controls, the cultures were incubated with the corresponding volume of the buffer without any peptide. The experiment was replicated three times. After 1 h, three ten-fold serial dilutions of the cultures were plated in triplicates on BHI media and incubated at 37°C overnight. The number of colony forming units on each plate was then recorded. Median lethal dose (LD_50_) was calculated for both *Ligand-Hecate* and *Hecate* using probit analysis (dose-response curve) for each microorganism.

### Genetic engineering of *K. lactis* to express recombinant proteins

The commercially available yeast-based protein expression system (*K. lactis*, New England Biolabs Inc., MA, USA) was genetically engineered to produce two strains to express and secrete two types of proteins: (a) a far red fluorescent protein, *mPlum* (Clontech Laboratories Inc., CA, USA), and (b) *Ligand-Hecate* fusion peptide. DNA sequences of *mPlum* and *Ligand-Hecate* were codon optimized for expression in *K. lactis* by GenScript Ltd., NJ, USA. The *mPlum* gene was amplified using primers (forward - 5′TTATGCTTCCGGCTCGTATG 3′and reverse - 5′AGGCCTATTATTTTTGACACCAGA3′). The *Ligand-Hecate* gene was amplified using primers (forward - 5′GTAAAACGACGGCCAGT3′and reverse -5′CAGGAAACAGCTATGAC3′). The amplified *mPlum* fragment was cloned into the *BamHI – EcoRI* site of pKLAC2 downstream of the *K. lactis* α-mating factor domain (α-MF) according to cloning strategy given in the instruction manual. Similarly, the amplified *Ligand-Hecate* fragment was cloned into *XhoI – NotI* site of pKLAC2 (Fig. S3A,B). For control, pKLAC2 without any foreign gene (*plasmid-only*) was included. All three constructs were cloned into competent *E. coli* cells (NEB # C2992, New England Biolabs Inc., MA, USA). Each vector was isolated and digested with a pair of respective restriction endonucleases to determine the presence of the insert.

All three pKLAC2 vectors (*mPlum*, *Ligand-Hecate* and *plasmid-only*) were linearized with *SacII* to generate the expression cassettes. The lineralized expression cassettes were introduced into competent *K. lactis* cells at the LAC4 locus according to the manufactures instructions. Cells of three yeast strains (*mPlum*, *Ligand-Hecate* and *plasmid-only*) were grown separately on yeast carbon base (YCB) agar medium containing 5 mM acetamide at 30°C for 2 days. Colonies of each strain were picked and resuspended in 2 ml YPGal medium and then incubated with shaking at 250 rpm for 2 days at 30°C. Cells of all the three yeast strains were harvested by centrifugation at 7000×g for 30 sec and the culture supernatants were transferred to fresh tubes.

Yeast cells with correct integration of the expression cassettes into the *K. lactis* genome were identified by PCR using the primers (Primer 1 -5′ACACACGTAAACGCGCTCGGT3′ and Primer 2 - 5′ATCATCCTTGTCAGCGAAAGC 3′) supplied with the *K*. *lactis* kit. Fresh colonies of each yeast strain were picked and resuspended in 25 µl of 1 M sorbitol containing 2 mg/ml lyticase. Cells were mixed by vortexing and incubated at 30°C. After 1 h, the lyticase-treated cells were lysed at 98°C for 10 min in a thermocyler. PCR was performed according to the *K*. *lactis* instruction manual. In case of each yeast strain, integration of the expression cassette at the LAC4 locus in the *K. lactis* genome resulted in amplification of a 2.4 kb product (the promoter region of the LAC4 locus) (Fig. S3C,D).

All harvested yeast strains (pelleted cells as well as culture supernatants) were tested for fluorescence under a fluorescent microscope (excitation – 590 nm and emission – 649 nm; Model: DMRxA2, Leica Microsystems Inc.) at 400 × magnification to confirm that red fluorescence was only produced by the *mPlum* yeast strain. Biological activity of the culture supernatants of yeast strains was determined against aerobic protozoa of the species *T*. *pyriformis*. Fifty microliter of the culture supernatants was incubated with 50 µl of *T. pyriformis* culture. After 24 h, live protozoa were counted using a Sedgewick-Rafter cell (Pyser-SGI Limited, Kent, UK) under a microscope (Model: DMLB, Leica Microsystems Inc.) at 200 × magnification.

### Termite feeding bioassays using genetically engineered yeast strains

All freshly harvested yeast strains were freeze-dried overnight using a lyophilizer. Freeze-drying does not affect the viability of yeast strains [56], [57]. Freeze-dried yeast strains were mixed separately at the rate of 7.5 mg with 1500 mg of α-cellulose powder and 3 ml water. Three disks of 0.5-cm thickness were punched out the mixture using a 1.5-cm-diameter cork borer. Each feeding experiment was set up in a Petri dish using a bait disk and 75 worker and 5 soldier termites. The Petri dishes were placed in a tray with moist paper towels and kept in an incubator at 26±2°C and 85% R.H. Each bait disk was hydrated with 300 µl autoclaved deionized water every 48 h. Termites were treated with four different bait disks containing: (a) *mPlum* yeast strain, (b) *Ligand-Hecate* yeast strain, (c) *plasmid-only* yeast strain (control), or (d) plain α-cellulose without any yeast (control). Three replicates were set up for each treatment. The whole experiment was repeated three times using three different termite colonies. The termite colonies were collected as explained above.

Fifteen worker guts from each replication were extirpated using sterile forceps at two- and three-week intervals of feeding and then divided into three groups of five guts for their use in three assays: (a) plating on *Kluyveromyces* differential medium, (b) testing for fluorescence, and (c) observing the status of gut protozoa.

(a) Plating on *Kluyveromyces* differential medium: A group of five guts (per replication) was homogenized in a microcentrifuge tube containing 500 µl of autoclaved deionized water. Three ten-fold serial dilutions of homogenized gut contents were prepared and plated in triplicates on *Kluyveromyces* differential medium [58]. Plates were incubated at 30°C for 48 h. Cells of *K. lactis* produced blue colonies on the medium due to the presence of X-Gal/IPTG. All remaining yeast species produced white, cream or pink color colonies [58]. Blue colonies were counted to assess uptake, survival and multiplication of yeast cells inside the termite guts.

(b) Testing for fluorescence: The gut contents were prepared from a group of five workers (per replication) as explained above and viewed under a fluorescent microscope (excitation – 590 nm and emission – 649 nm; Model: DMRxA2, Leica Microsystems Inc.) at 400 × magnification for the presence of *mPlum* fluorescence in yeast strains.

(c) Observing status of gut protozoa: Five worker hindguts (per replication) were cut open in sparged Trager U media on a glass slide using fine probes in an anaerobic glovebox and the status of the gut protozoa was checked under both stereo (Model: MZ16, Leica Microsystems) and (Model: DMLB, Leica Microsystems) compound microscopes at 50 and 200 × magnification, respectively.

After five weeks of termite feeding, dry weight of bait consumed was calculated for comparison among the treatments. To determine bait dry weight, an additional 10 bait disks from each treatment (*mPlum*, *Ligand*-*Hecate*, *plasmid*-*only* and control) were weighed individually (disk fresh weight) before they were put into an oven at 50±5°C. After 48 h, these bait disks were reweighed individually (disk dry weight). A dry/fresh weight ratio was calculated for each bait disk and averaged over the 10 disks. The bait fresh disk from each treatment was weighed prior to the start of the feeding experiment, and dry weight was computed by multiplying with the corresponding average dry/fresh weight ratio. After five weeks of exposure to termite feeding, the bait disks were dried in the oven for 48 h at the same temperature. The dry weight of bait consumed was calculated as the difference between initial and final dry weights [59].

### Testing transfer of the genetically engineered yeast to other colony members

Following confirmation of *mplum* yeast strain in the termite gut (after 2 weeks of feeding on the bait containing *mplum* yeast strain), the remaining termites (donors) were mixed with an equal number of workers (recipients) from the same colony that were fed on a bait of α-cellulose without yeast. The recipient termites were marked red by feeding them with filter paper containing 1% (w/w, 6.0 mg stain per paper) Sudan Red 7B to distinguish between donors and recipients termites [15]. The mixed termites were fed on plain α-cellulose bait without any yeast in a Petri dish. The whole experiment was carried out with three replicates and was repeated twice using two different termite colonies. Termites were collected as explained above.

The guts of five donors and five recipients were extirpated at two- and four-week intervals of feeding and homogenized in water. The homogenate was spread on *Kluyveromyces* Differential Medium as described above to quantify the presence of yeast in donors and recipients and confirm the transfer of yeast to the recipients by counting the number of blue colonies. Since the yeast strain contained the *mPlum* gene, we also confirmed transfer and gene expression of the yeast by viewing the gut homogenate under a fluorescent microscope as described above.

### Statistical analyses

The dose-response data on *Ligand-Hecate* and *Hecate* against *E*. *coli*, *P*. *termitis*, and *K*. *lactis* was subjected to probit analysis and the values obtained for mean lethal dose (LD_50_) were compared within each microorganism using t-test (JMP software, SAS Institute). The data on: (1) the number alive protozoa *T. pyriformis* in biological activity assay using the culture supernatants of yeast strains, (2) the number of yeast cells (CFU) per termite gut in yeast feeding assays, and (3) diet consumption in yeast feeding assay were analyzed using analysis of variance. Then, Tukey's honestly significant difference (HSD) test with a significance level of α = 0.05 was used for post hoc means separation (JMP software, SAS Institute).

## Supporting Information

Figure S1 **Identification and construction of ligands that bind to protozoa living in the hindgut of the Formosan subterranean termite,** ***Coptotermes formosanus*** **.** SEM images of the three species of protozoa: (*A*) *Pseudotrichonympha grassii*, (*B*) *Holomastigotoides hartmanni*, and (*C*) *Spirotrichonympha leidyi*. (*D*) Cross section of the three species of protozoa. (*E*) SEM and (*F*) TEM images of isolated plasma membrane from the protozoa. (*G*) Scheme explaining panning of isolated plasma membrane with a phage library consists of linear heptapeptides (Ph.D. 7). (*H, I*) Two selected ligands (*Ligand-1* and *Ligand-2*) attached to a fluorophore *EDANS* (5-((2-Aminoethyl) amino) naphthalene-1-sulfonic acid). (*J*) Fusion peptide consisting of *Ligand-1* and *Hecate*.
(TIF)

Figure S2 **Visualization of untreated gut protozoa of the Formosan subterranean termite,** ***Coptotermes formosanus*** **under fluorescence microscope.** (*A, B, C*) Superimposed fluorescent (excitation  =  341 nm, emission  =  471 nm) and differential interference contrast (DIC) exposures of *Pseudotrichonympha grassii*, *Holomastigotoides hartmanni* and *Spirotrichonympha leidyi*, respectively. Phagocytosed wood particles within the protozoa cytoplasm show some patchy autofluorescence.
(TIF)

Figure S3 **Genetic engineering of** ***Kluyveromyces lactis*** **yeast to produce two strains** ***mPlum*** **and** ***Ligand-Hecate.*** (*A*) The pKLAC2 expression vector. (*B*) Cloning strategy for *mPlum* and *Ligand-Hecate* into pKLAC2. (*C*) Genomic integration of two expression cassettes, *mPlum* and *Ligand-Hecate* in the *K. lactis* genome. Vector pKLAC2 containing either *mPlum* or *Ligand-Hecate* was digested with SacII and introduced into *K. lactis* cells. The 5′ PLAC4 and 3′ PLAC4 sequences directed insertion of the cassette into the promoter region of the LAC4 locus in the *K. lactis* genome. (*D*) Genetically engineered *K. lactis* cells in which the expression cassette had correctly integrated into the *K. lactis* genome were identified by PCR using supplied Integration Primers 1 and 2 to amplify a 2.4 kb product (the promoter region of the LAC4 locus).
(TIF)

Table S1 **Protozoa recognition peptides identified using phage display libraries.** Two heptapeptide sequences (shown in red) were selected to synthesized two ligands, *Ligand-1* and *Ligand-2*, respectively.
(DOCX)

Table S2 **ANOVA of the number of yeast CFU per termite gut at two and three weeks of ingesting α-cellulose diets.**
(DOCX)

Table S3 **ANOVA of the number of** ***mPlum*** **yeast CFU per termite gut at two and four weeks after combining the donors and recipients.**
(DOCX)
