# Supplementary figures and images for: Protozoacidal Trojan-Horse: Use of a Ligand-Lytic Peptide for Selective Destruction of Symbiotic Protozoa within Termite Guts
Source: PLoS One. 2014 Sep 8;9(9):e106199. doi: 10.1371/journal.pone.0106199 (PMC4157778; doi:10.1371/journal.pone.0106199)

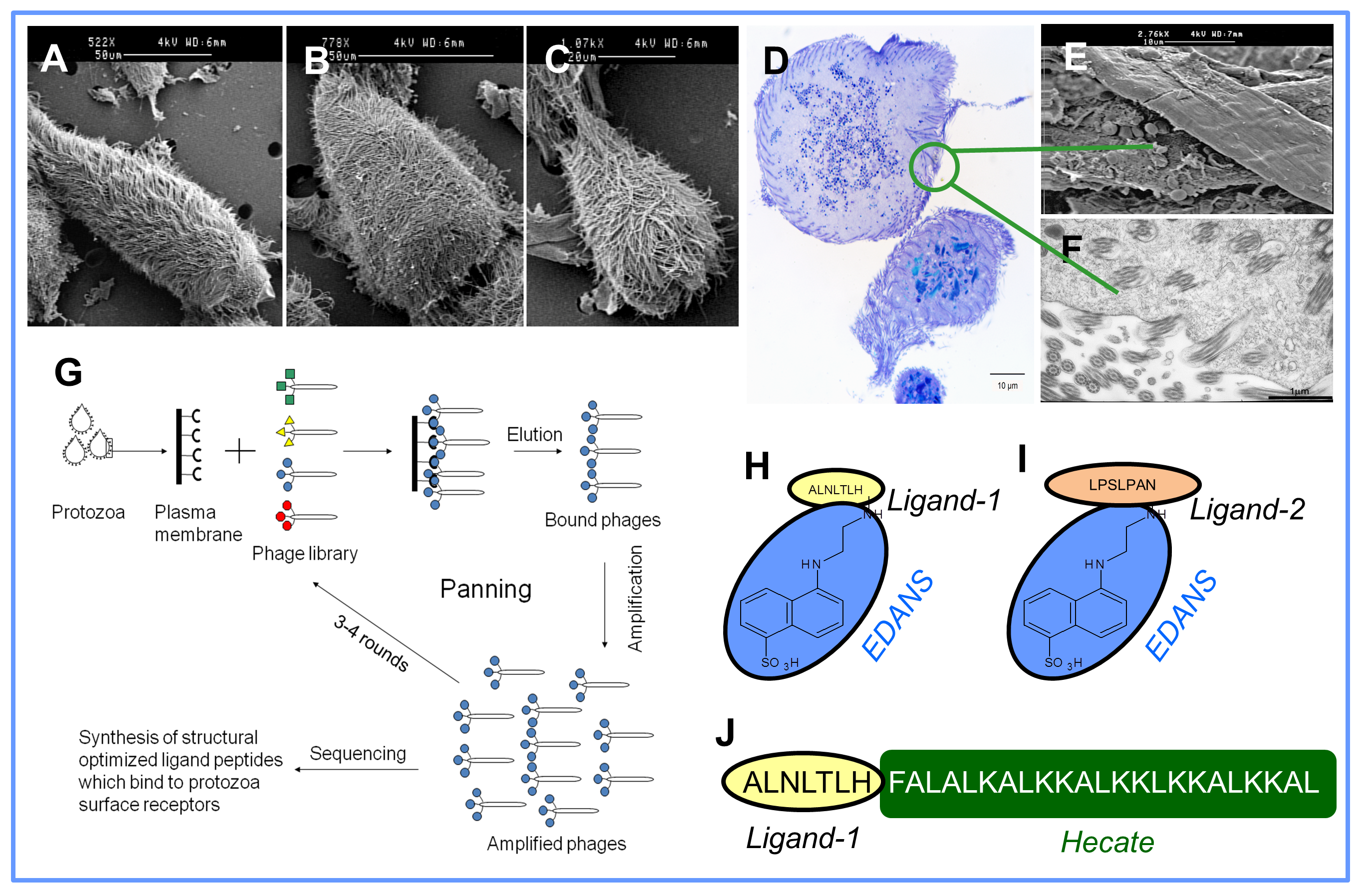

Supplement: Figure S1 — Identification and construction of ligands that bind to protozoa living in the hindgut of the Formosan subterranean termite, Coptotermes formosanus . SEM images of the three species of protozoa: (A) Pseudotrichonympha grassii, (B) Holomastigotoides hartmanni, and (C) Spirotrichonympha leidyi. (D) Cross section of the three species of protozoa. (E) SEM and (F) TEM images of isolated plasma membrane from the protozoa. (G) Scheme explaining panning of isolated plasma membrane with a phage library consists of linear heptapeptides (Ph.D. 7). (H, I) Two selected ligands (Ligand-1 and Ligand-2) attached to a fluorophore EDANS (5-((2-Aminoethyl) amino) naphthalene-1-sulfonic acid). (J) Fusion peptide consisting of Ligand-1 and Hecate. (TIF) [file pone.0106199.s001.tif]

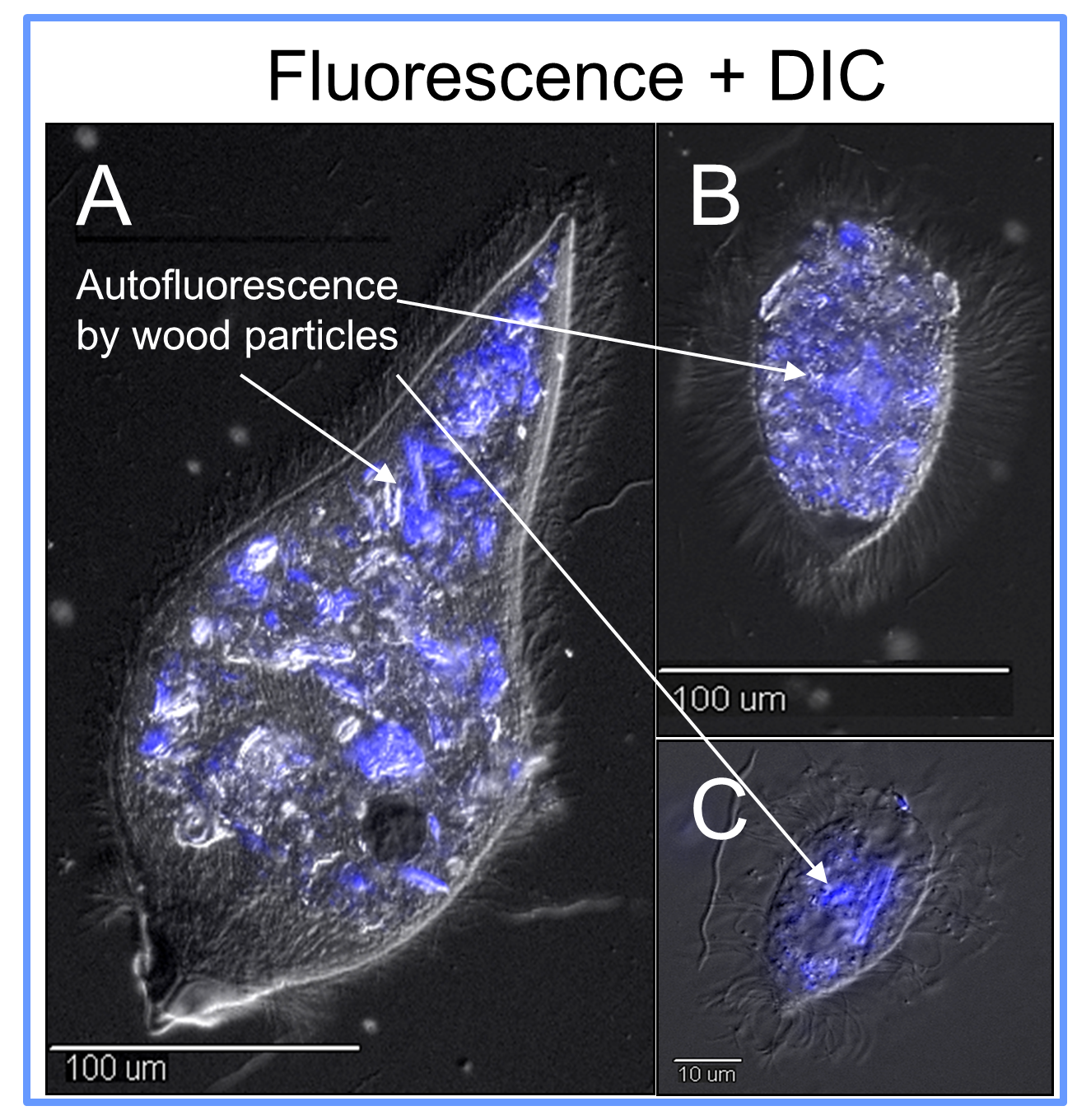

Supplement: Figure S2 — Visualization of untreated gut protozoa of the Formosan subterranean termite, Coptotermes formosanus under fluorescence microscope. (A, B, C) Superimposed fluorescent (excitation = 341 nm, emission = 471 nm) and differential interference contrast (DIC) exposures of Pseudotrichonympha grassii, Holomastigotoides hartmanni and Spirotrichonympha leidyi, respectively. Phagocytosed wood particles within the protozoa cytoplasm show some patchy autofluorescence. (TIF) [file pone.0106199.s002.tif]

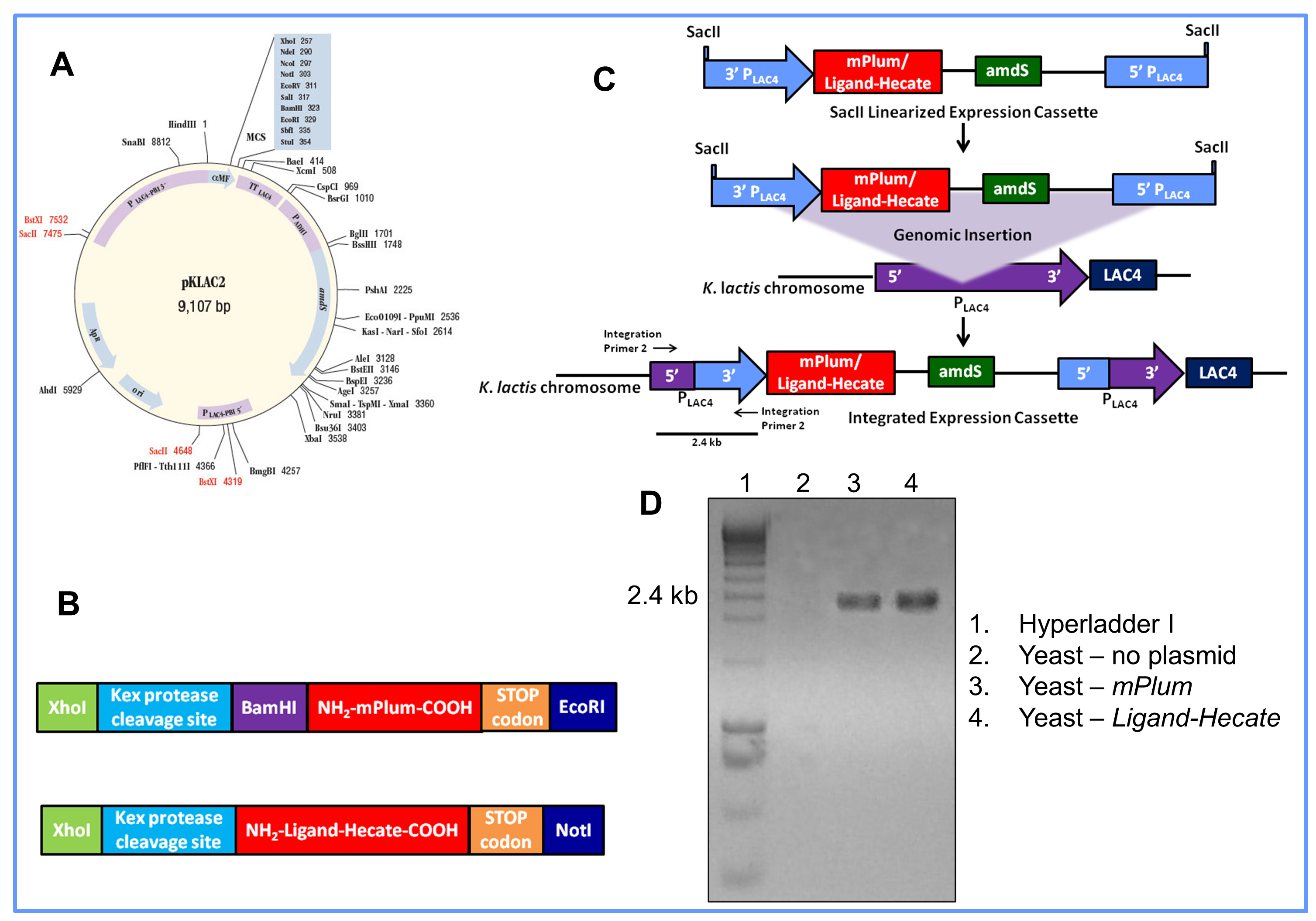

Supplement: Figure S3 — Genetic engineering of Kluyveromyces lactis yeast to produce two strains mPlum and Ligand-Hecate. (A) The pKLAC2 expression vector. (B) Cloning strategy for mPlum and Ligand-Hecate into pKLAC2. (C) Genomic integration of two expression cassettes, mPlum and Ligand-Hecate in the K. lactis genome. Vector pKLAC2 containing either mPlum or Ligand-Hecate was digested with SacII and introduced into K. lactis cells. The 5′ PLAC4 and 3′ PLAC4 sequences directed insertion of the cassette into the promoter region of the LAC4 locus in the K. lactis genome. (D) Genetically engineered K. lactis cells in which the expression cassette had correctly integrated into the K. lactis genome were identified by PCR using supplied Integration Primers 1 and 2 to amplify a 2.4 kb product (the promoter region of the LAC4 locus). (TIF) [file pone.0106199.s003.tif]
