# Supplementary material for: Protozoacidal Trojan-Horse: Use of a Ligand-Lytic Peptide for Selective Destruction of Symbiotic Protozoa within Termite Guts
Source: PLoS One. 2014 Sep 8;9(9):e106199. doi: 10.1371/journal.pone.0106199 (PMC4157778; doi:10.1371/journal.pone.0106199)
Supplement: Table S1 — Protozoa recognition peptides identified using phage display libraries. Two heptapeptide sequences (shown in red) were selected to synthesized two ligands, Ligand-1 and Ligand-2, respectively. (DOCX) [file pone.0106199.s004.docx]

**Table S1**. **Protozoa recognition peptides identified using phage display libraries.** Two heptapeptide sequences (shown in red) were selected to synthesized two ligands, *Ligand-1* and *Ligand-2*, respectively.

| Peptide sequence | Number of hydrophobic residues | Percent hydrophobicity | Match | Percent identity | Accession Number |
| --- | --- | --- | --- | --- | --- |
| ALNLTLH | 4 | 57 | Variant surface glycoprotein | 100 | [Q57WM6](http://www.uniprot.org/uniprot/Q57WM6) |
| LPSLPAN | 4 | 57 | Tlr0055 membrane protein | 100 | [Q8DMQ6](http://www.uniprot.org/uniprot/Q8DMQ6) |
| DGTIGLR | 4 | 57 | Ras-related protein Rab-6C | 100 | [Q9H0N0](http://www.uniprot.org/uniprot/Q9H0N0) |
| SVYSPSP | 3 | 43 | Putative uncharacterized protein | 100 | [G5ACT9](http://www.uniprot.org/uniprot/G5ACT9) |
| IPPAPPH | 5 | 71 | Putative uncharacterized protein | 100 | [A5ASC8](http://www.uniprot.org/uniprot/A5ASC8) |
| QLAPITG | 5 | 71 | Putative outer membrane receptor for ferric iron uptake | 100 | [Q3IFM9](http://www.uniprot.org/uniprot/Q3IFM9) |
| ASYSGTA | 3 | 43 | Phosphate/phosphonate ABC transporter periplasmic substrate-binding protein | 100 | [B9LSD6](http://www.uniprot.org/uniprot/B9LSD6) |
| SSPYYIN | 2 | 29 | GJ23376 | 100 | [B4LY23](http://www.uniprot.org/uniprot/B4LY23) |
| LYAVPNT | 4 | 57 | Cyclic peptide transporter | 100 | [A5FI44](http://www.uniprot.org/uniprot/A5FI44) |
| VPRPLLL | 6 | 86 | Binding-protein-dependent transport systems inner membrane component precursor | 100 | [C6WL46](http://www.uniprot.org/uniprot/C6WL46) |
| QFSFEHQ | 2 | 29 | No match | - |  |
| WPSHGMS | 4 | 57 | No match | - |  |
| KHNYPAH | 2 | 29 | No match | - |  |
| VSLTLMQ | 4 | 57 | No match | - |  |
| EEMSFLL | 4 | 57 | No match | - |  |
| TIQDHSR | 1 | 14 | No match | - |  |
| TFIQPVS | 4 | 57 | No match | - |  |
| SLVNSVP | 4 | 57 | No match | - |  |
| SLRAQQA | 3 | 43 | No match | - |  |
