# Supplementary material for: Protozoacidal Trojan-Horse: Use of a Ligand-Lytic Peptide for Selective Destruction of Symbiotic Protozoa within Termite Guts
Source: PLoS One. 2014 Sep 8;9(9):e106199. doi: 10.1371/journal.pone.0106199 (PMC4157778; doi:10.1371/journal.pone.0106199)
Supplement: Table S2 — ANOVA of the number of yeast CFU per termite gut at two and three weeks of ingesting α-cellulose diets. (DOCX) [file pone.0106199.s005.docx]

**Table S2.** **ANOVA of the number of yeast CFU per termite gut at two and three weeks of ingesting α-cellulose diets.**

**Analysis of Variance**

| **Source** | **DF** | **Sum of Squares** | **Mean Square** | **F Ratio** | **Prob > F** |
| --- | --- | --- | --- | --- | --- |
| Model | 23 | 2.09E+10 | 9.11E+08 | 4.092709 | <.0001 |
| Error | 48 | 1.07E+10 | 2.23E+08 |  |  |
| C. Total | 71 | 3.16E+10 |  |  |  |

**Effect Tests**

| **Source** | **DF** | **Sum of Squares** | **F Ratio** | **Prob > F** |
| --- | --- | --- | --- | --- |
| Colony | 2 | 5.78E+08 | 1.298933 | 0.2822 |
| Diet | 3 | 7.25E+09 | 10.85877 | <.0001 |
| Time | 3 | 9.31E+09 | 41.81566 | <.0001 |
| Colony*Diet | 6 | 2.26E+08 | 0.1693 | 0.9838 |
| Colony*Time | 2 | 2.25E+08 | 0.505903 | 0.6061 |
| Diet*Time | 3 | 3.24E+09 | 4.848157 | 0.0050 |
| Colony*Diet*Time | 6 | 1.27E+08 | 0.095069 | 0.9966 |
