# Supplementary material for: Protozoacidal Trojan-Horse: Use of a Ligand-Lytic Peptide for Selective Destruction of Symbiotic Protozoa within Termite Guts
Source: PLoS One. 2014 Sep 8;9(9):e106199. doi: 10.1371/journal.pone.0106199 (PMC4157778; doi:10.1371/journal.pone.0106199)
Supplement: Table S3 — ANOVA of the number of mPlum yeast CFU per termite gut at two and four weeks after combining the donors and recipients. (DOCX) [file pone.0106199.s006.docx]

**Table S3.** **ANOVA of the number of *mPlum* yeast CFU per termite gut at two and four weeks after combining the donors and recipients.**

**Analysis of Variance**

| **Source** | **DF** | **Sum of Squares** | **Mean Square** | **F Ratio** | **Prob > F** |
| --- | --- | --- | --- | --- | --- |
| Model | 7 | 2.83E+09 | 4.04E+08 | 10.77612 | <.0001 |
| Error | 16 | 6.00E+08 | 37522200 |  |  |
| C. Total | 23 | 3.43E+09 |  |  |  |

**Effect Tests**

| **Source** | **DF** | **Sum of Squares** | **F Ratio** | **Prob > F** |
| --- | --- | --- | --- | --- |
| Organism | 1 | 2.06E+09 | 5.48E+01 | <.0001 |
| Time | 1 | 4.45E+08 | 1.19E+01 | 0.0033 |
| Colony | 1 | 6283267 | 0.167455 | 0.6878 |
| Organism*Time | 1 | 3.1E+08 | 8.25116 | 0.0111 |
| Organism*Colony | 1 | 10296600 | 0.274414 | 0.6076 |
| Time*Colony | 1 | 1251267 | 0.033347 | 0.8574 |
| Organism*Time*Colony | 1 | 1771267 | 0.047206 | 0.8307 |
